# Editorial: Advanced functional nanomaterials for diagnosis, bioimaging, drug delivery and therapeutics

**DOI:** 10.3389/fmolb.2024.1399695

**Published:** 2024-03-25

**Authors:** Eudald Casals, Shunbo Li, Zhiyu Jia, Gregori Casals, Muling Zeng

**Affiliations:** ^1^ School of Biotechnology and Health Sciences, Wuyi University, Jiangmen, China; ^2^ Key Laboratory of Optoelectronic Technology and Systems, Ministry of Education and Key Disciplines Laboratory of Novel Micro-Nano Devices and System Technology, College of Optoelectronic Engineering, Chongqing University, Chongqing, China; ^3^ Key Laboratory of Cluster Science, Ministry of Education of China, Beijing Key Laboratory of Photoelectronic/Electrophotonic Conversion Materials, School of Chemistry and Chemical Engineering, Beijing Institute of Technology, Beijing, China; ^4^ Service of Biochemistry and Molecular Genetics, Hospital Clinic Universitari and the August Pi i Sunyer Biomedical Research Institute (IDIBAPS), Barcelona, Spain; ^5^ Liver and Digestive Diseases Networking Biomedical Research Centre (CIBEREHD), Madrid, Spain; ^6^ Department of Fundamental Care and Medical-Surgical Nursing, Faculty of Medicine and Health Sciences, University of Barcelona, Barcelona, Spain

**Keywords:** advanced functional and healthcare materials, drug delievery, bioimaging, diagnosis, nanomedicine

In the healthcare domain, nanomaterials play a pivotal role in simultaneous disease detection and treatment, addressing conditions such as cancer (Casals et al., 2017), neurological disorders (Liu et al., 2021), cardiovascular diseases (Smith and Edelman, 2023) and inflammatory processes (Casals et al., 2020), among others. The emergence of theranostic nanomedicines offers flexible platforms tailored for personalized treatments. In addition, owing to their inherent detectability by many biomedical imaging techniques, combined with the robust nanostructures developed in recent years providing shelter and/or stability to incorporated active molecules, nanomaterials are perfectly suited for combined diagnostics and therapy (Zeng et al., 2021) and some even exhibit intrinsic therapeutic actions (Prabhakar et al., 2021), such as phototherapy and anti-inflammatory treatments (Parra-Robert et al., 2019). However, the application of nanomaterials in health-related domains has not been without its challenges (Ghorbani et al., 2021). The inherent complexity of physiological processes necessitates high standards for the development and deployment of these advanced functional nanomaterials, demanding innovative solutions to ensure efficacy, safety, and compatibility within intricate biological systems.

A significant contribution to the scenario described above is the review article by Long et al. which addressed the rising concern of brain metastases (BrM) and associated challenges with current breakthrough therapies enabled by nanomaterials. The article first discussed how malignant tumor cells manipulate the brain microenvironment, transitioning it from anti-tumoral to pro-tumoral. It then compared the characteristics of the brain microenvironment in BrM with other sites or primary tumors. Subsequently, the article evaluated microenvironment-targeted therapies enabled by nanomaterials, showcasing promise in overcoming drug resistance and low blood-brain barrier permeability. The article suggested how these diverse therapies could lead to improved outcomes for patients with secondary brain tumors.

Continuing with novel therapies to interact with complex pathophysiological processes, the original research article by Xu et al. introduced a supramolecular strategy to extend the *in vivo* half-life and improve tumor targeting of therapeutic proteins. The study focused on the fusion of trichosanthin (TCS) with the self-assembling protein Sup35p prion domain (Sup35), resulting in the formation of uniform spherical TCS-Sup35 nanoparticles (TCS-Sup35 NP) with pH-responsive characteristics. TCS-Sup35 NP retained the bioactivity of TCS and exhibited a significantly longer *in vivo* half-life than native TCS. In a tumor-bearing mouse model, TCS-Sup35 NP showed improved tumor accumulation and antitumor activity without detectable systemic toxicity, presenting a promising solution for enhancing the pharmacological performance of therapeutic proteins with short circulation half-lives.

Furthermore, addressing unmet clinical needs poses challenges for the application of nanomaterials, including intricate synthesis processes of sophisticated nanoparticles and Research Topic with drug delivery nanocarriers, such as slow release in physiological conditions. The review article by Zhang et al. explored cutting-edge applications of DNA origami in antitumor drug delivery. DNA origami, a revolutionary self-assembly technique, creates precise nanostructures with lower biotoxicity, increased stability, and superior adaptability. The review discussed principles, design strategies, and the latest research achievements of DNA origami in anti-tumor drug delivery, emphasizing the potential to reduce side effects and enhance therapy success through precise, targeted, and multifunctional drug delivery systems. The article also explored the use of DNA tetrahedra in addressing drug delivery challenges in cancer therapy.

Importantly, nanomaterials involved in therapeutics or imaging must address biocompatibility and biosafety Research Topic within the human body. The original research article by Liu et al. focused on using Rhodobacter sphaeroides as a biological model to study the ecotoxicity of 1-alkyl-3-methylimidazolium bromide ([Cnmim]Br), an ionic liquid. The study correlated the inhibition of bacterial growth with the alkyl chain length and investigated morphological changes, including cell membrane perforation. The research utilized electrochromic absorption bands to demonstrate linear correlations with the alkyl chain length. Moreover, it observed effects on ATP synthesis and antioxidant enzyme activity. The findings proposed Rhodobacter sphaeroides as a valuable model for monitoring ecotoxicity and understanding the mechanisms of ionic liquid toxicity. Importantly, this study addressed both safety aspects and demonstrated improved specificity and sensitivity, vital for diagnostic applications.

In summary, this Research Topic invited global contributions from professionals in industry, academia, and research institutions, with the aim of accelerating advancements in advanced functional nanomaterials for more effective diagnosis, bioimaging, drug delivery, and therapeutic interventions. This collaborative effort has explored and contributed to framing recent advancements and challenges in the broad intersection of nanotechnology and healthcare.
